# Supplementary material for: Crystallisation Phenomena of In2O3:H Films
Source: Materials (Basel). 2019 Jan 15;12(2):266. doi: 10.3390/ma12020266 (PMC6356562; doi:10.3390/ma12020266)
Supplement: Supplementary file 1 [file materials-12-00266-s001.pdf]

materials

Supplementary information

Crystallisation Phenomena of In_2O_3:H Films

Ruslan Muydinov [1,*], Alexander Steigert [2], Markus Wollgarten [3], Paweł Piotr Michałowski [4], Ulrike Bloeck [3], Andreas Pflug [5], Darja Erfurt [6], Reiner Klenk [6], Stefan Körner [1], Iver Lauermann [6] and Bernd Szyszka [1]

[1] Institute of Semiconducting- and High-Frequency Technologies, Technical University Berlin, Einsteinufer 25, 10587 Berlin, Germany; s.koerner@tu-berlin.de (S.K.); bernd.szyszka@tu-berlin.de (B.S.)
[2] Institute for Nanospectroscopy, Helmholtz-Zentrum Berlin, Albert-Einstein-Str. 15, 12489 Berlin, Germany; alexander.steigert@helmholtz-berlin.de
[3] Department of Nanoscale Structures and Microscopic Analysis, Helmholtz-Zentrum Berlin, Hahn-Meitner-Platz 1, 14109 Berlin, Germany; wollgarten@helmholtz-berlin.de (M.W.); bloeck@helmholtz-berlin.de (U.B.)
[4] Institute of Electronic Materials Technology, Wolczynska Str. 133, 01919 Warsaw, Poland; Pawel.Michalowski@itme.edu.pl
[5] Fraunhofer Institute for Surface Engineering and Thin Films IST, Bienroder Weg 54e, 38108 Braunschweig, Germany; Andreas.Pflug@ist.fraunhofer.de
[6] PVcomB, Helmholtz-Zentrum Berlin, Schwarzschildstr. 3, 12489 Berlin, Germany; darja.erfurt@helmholtz-berlin.de (D.E.); klenk@helmholtz-berlin.de (R.K.); iver.lauermann@helmholtz-berlin.de (I.L.)
* Correspondence: ruslan.muydinov@tu-berlin.de

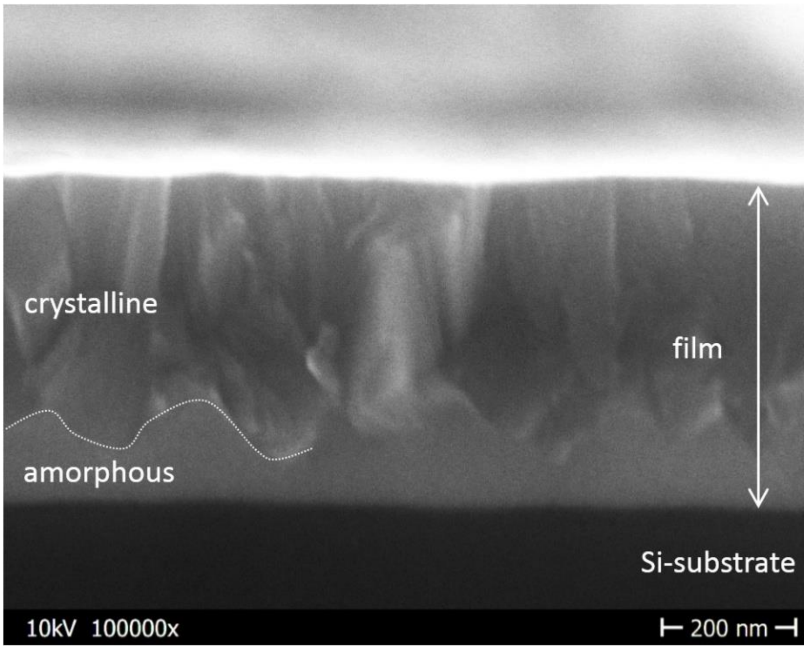

Figure S1. SEM cross-section of the RF sputtered (p_{tot} = 0.5 Pa) 500 nm In_2O_3:H_2O film obtained on Si-substrate.

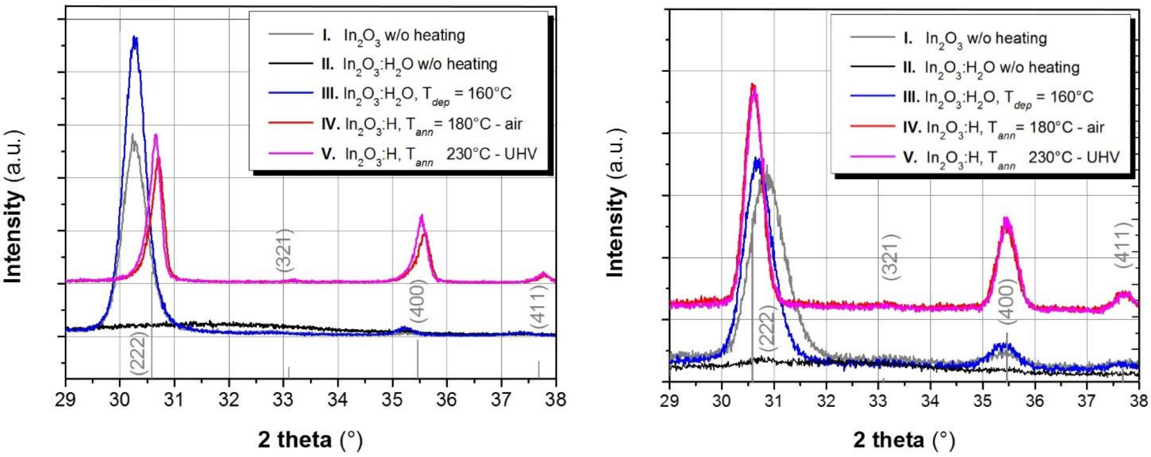

(**a**) (**b**)

Figure S2. XRD patterns for ~150 nm In_2O_3 films deposited (RF-sputtering, p_{tot} = 0.5 Pa) on glass: comparison of crystallisation conditions. Roman numerals correspond to the film states discussed in the text. Diffraction patterns were acquired using detector-scanning at grazing incidence in the *out-of-plane* (**a**) and *in-plane* (**b**) modes.

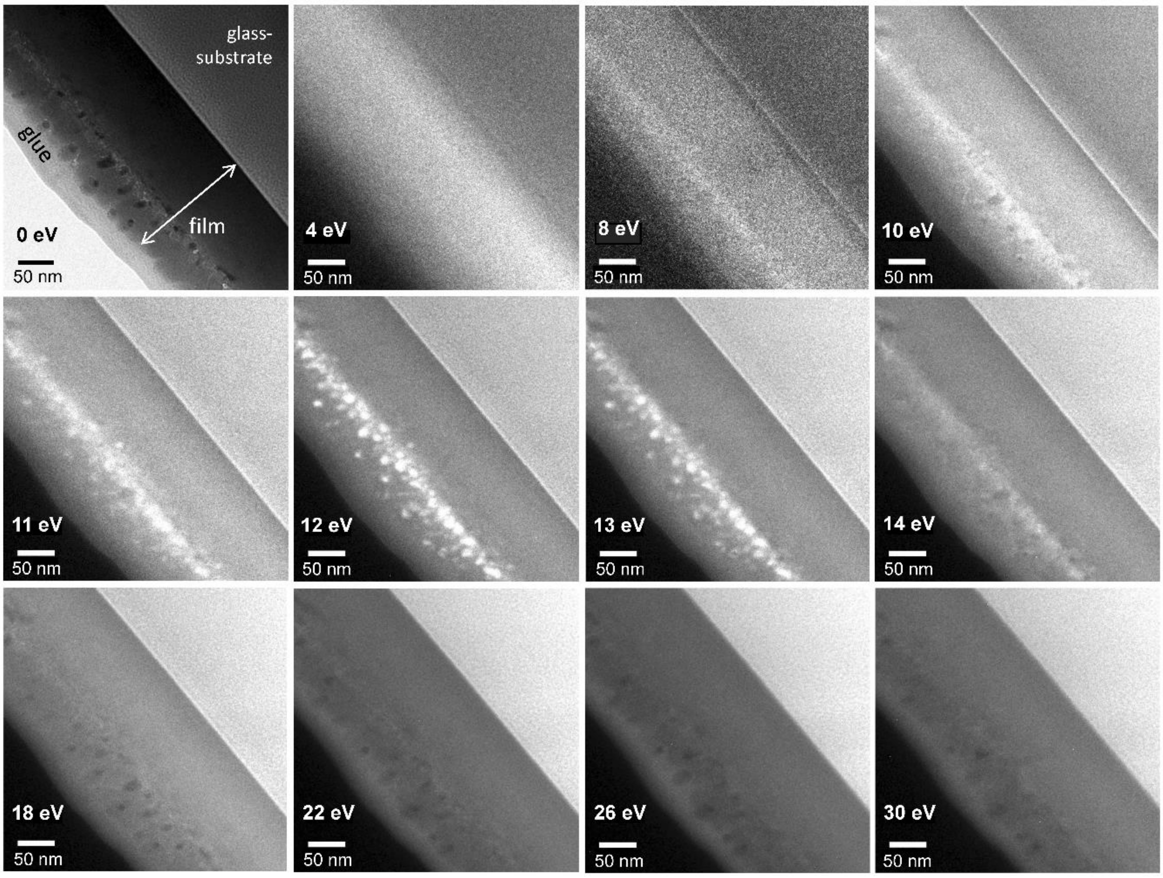

Figure S3. TEM images obtained using energy filter. The set energy is marked on each image.

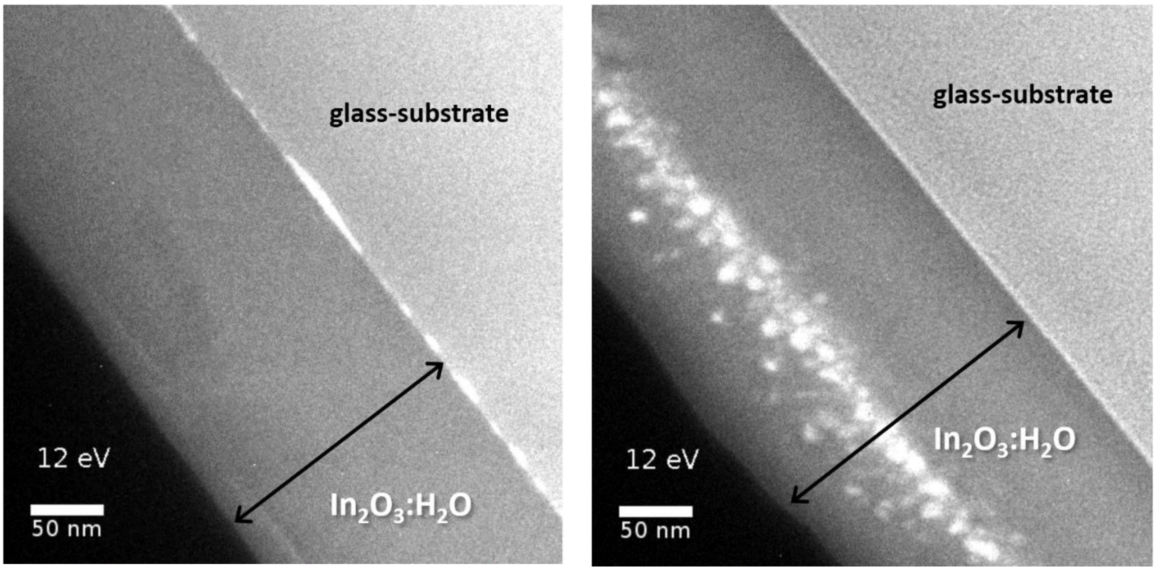

(**a**) (**b**)

Figure S4. Cross-sectional TEM images acquired with electrons having 12 eV energy loss on the sample. The bright areas correspond therefore to metallic indium in as-deposited In_2O_3:H_2O film. Two types of indium segregation: on the film/glass interface (**a**) and within the bulk of the film (**b**) are observed.

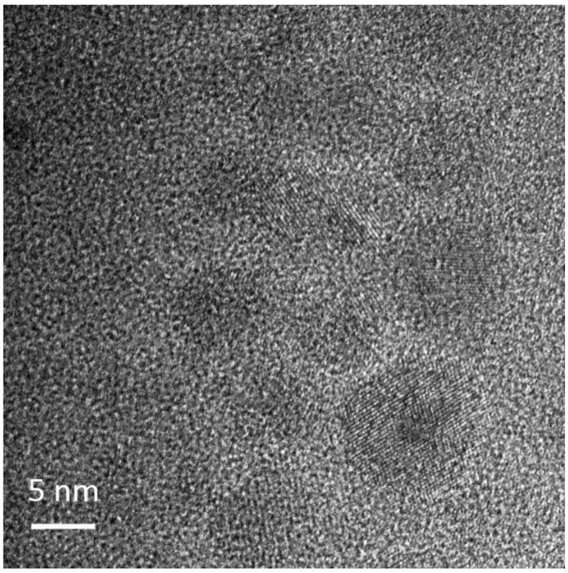

Figure S5. TEM image of metallic indium nanoparticles released in In_2O_3:H_2O matrix. The lattice fringe contrast observed reveals their crystalline state.

We present here many TEM and EELS figures which demonstrate variable thickness and crystalline state (compare Figure S4a,b) and this needs to be commented. The sample presented in the Figures 2, 3 and S3–S5 is the same, however we investigated two lamella. First lamella was unexpectedly partially crystallized during TEM investigation (Figure 2). Then we provided better heat dissipation via specimen holder in another TEM system with EELS. Figures 3, S3, S4b and S5 were obtained from the different places of the lamella No.2. Figure S2a shows the remaining amorphous part of the lamella No.1. Evidently, the thickness of the film is somewhat different in these two lamella. Thicker film (lamella No.2) is also partially crystallised from the top. This effect is similar to the one discussed on the Figure S1. We suggest that the difference between lamella is caused by the magnetron. Namely, the regions of the film being directly opposite to the racetracks of electrons should be heated by plasma more impactful. An example of such inhomogeneity is presented in our previous paper [Reference 13].
